# Optimal timing of cancer treatments: a call for emerging evidence from clinical trials and real-world studies

**DOI:** 10.1038/s41416-025-03030-4

**Published:** 2025-04-23

**Authors:** Jianrong Zhang, Rebecca Venchiarutti, Xiaofei Wang, Qihua He

**Affiliations:** 1 Melbourne Medical School & Centre for Cancer Research, Faculty of Medicine, Dentistry and Health Sciences, University of Melbourne, Melbourne, VIC Australia; 2 Victorian Comprehensive Cancer Centre, Melbourne, VIC Australia; 3 Victorian Cancer Registry, Cancer Council Victoria, Melbourne, VIC Australia; 4 School of Health & Wellbeing, College of Medical, Veterinary & Life Sciences, University of Glasgow, Glasgow, UK; 5 Department of Head and Neck Surgery, Chris O’Brien Lifehouse, Camperdown, NSW Australia; 6 Sydney School of Public Health, Faculty of Medicine and Health, University of Sydney, Camperdown, NSW Australia; 7 Department of Biostatistics & Bioinformatics, Duke University School of Medicine, Durham, NC USA; 8 Alliance Statistics and Data Management Center, Duke University, Durham, NC USA; 9 Department of Thoracic Surgery and Oncology, First Affiliated Hospital of Guangzhou Medical University, Guangzhou, Guangdong China; 10 State Key Laboratory of Respiratory Disease, National Clinical Research Centre for Respiratory Disease, Guangzhou Institute of Respiratory Health, Guangzhou, Guangdong China

**Keywords:** Cancer therapy, Outcomes research, Epidemiology, Therapeutics

## Abstract

Cancer treatment has entered the era of personalised or precision medicine. Biomarker-driven therapies provide improved treatment efficacy and manageable toxicity profiles compared to systemic standard-of-care therapies. They also drive the development of combining non-surgical treatments, extending indications to early-stage tumours and further refining treatment lines with more precise options. The current treatment landscape, however, has introduced a complexity of approaches to cancer treatment, including the optimal timing of when to initiate and discontinue these treatments. Of note, treatment timing usually lacks evaluation in clinical trials and can be variable in real-world settings due to the impacts of medical, healthcare, and social factors. Given that more patients can benefit from multi-modality strategies, a better understanding of the prognostic impact of treatment-to-treatment intervals (TTIs) – the intervals between combined treatments and between treatment lines – is needed. Studies for this purpose can rely on existing trial and real-world data and be context-specific for treatment options, therapeutic settings, cancer types and biomarkers, healthcare settings or systems. This perspective article calls for emerging evidence of the optimal timing of cancer treatments. We anticipate that new studies on the optimal timing will bring new insights into how to better use cancer treatments, further improving treatment efficacy.

Early detection and timely treatment are essential to ensure optimal survival and avoid long-term morbidities associated with complex therapies for advanced cancers. However, delayed diagnosis and long wait times for treatment remain significant healthcare problems globally, risking the progression of disease to more advanced stages and potentially shifting the treatment intent from curative to palliative [1].

Cancer treatment has entered the era of personalised or precision medicine. Cancer cells and the tumour microenvironment can be targeted by advanced therapies like targeted therapies and immunotherapies based on the expression of biomarkers, providing superior treatment efficacy and manageable toxicity profiles to systemic standard-of-care therapies [2, 3]. Such features drive progressive development of existing treatment options: combining non-surgical treatment options, extending indications to earlier-stage, resectable cancers in the adjuvant or neoadjuvant settings, and further refining treatment lines with more precise options [1–3]. Chemotherapy and radiotherapy continue to evolve, improving the above strategies for better outcomes [2, 3].

## Optimal timing of cancer treatments

With the increasing complexity of approaches to cancer treatment, especially biomarker-driven therapies, the impact of treatment timing on prognosis becomes a pressing question to answer. Despite randomised clinical trials (RCTs) being the gold standard of evidence-based medicine, few oncological RCTs have been designed to investigate the optimal timing of cancer treatments before trial implementation. During implementation, trialists administer treatments to eligible patients; especially for adjuvant treatments, eligible patients can be selected given that they have successfully tolerated the previous treatment. Therefore, they commonly miss pre-specified time intervals between the treatments and lack an evaluation of their associations with patient outcomes. Practice guidelines and expert consensuses do not often suggest the optimal timing of treatment uses [4]. Some do [5, 6]; for example, no more than six weeks for postoperative radiation therapy (PORT) for surgically managed head and neck squamous cell carcinoma (HNSCC) [5] and surgery within 3–6 weeks of neoadjuvant treatment for early-stage non-small-cell lung cancer (NSCLC) [6]. However, at the same time, they claim no or low level of evidence to support the specific time windows [6]. Even with these practice guidelines, real-world studies have found that many patients do not receive treatment within the optimal timeframes (e.g., >50% of people with HNSCC do not receive PORT within the optimal timeframe) [7–9]. Selecting the time points for treatment initiation and discontinuation may have been arbitrary.

### Treatment-to-treatment interval

One key focus is the treatment-to-treatment interval (TTI) between multiple modalities within one single treatment strategy (e.g., the time between neoadjuvant immunotherapy [e.g., ipilimumab, nivolumab or both] and surgery for clinical stage III melanoma [3]), and between treatment lines (e.g., the time between first-line first-generation epidermal growth factor receptor [EGFR] tyrosine kinase inhibitor [TKI] and second-line third-generation EGFR TKI for advanced NSCLC [2]). Studies should be conducted to determine optimal TTIs so that patients will not risk poorer survival or quality of life.

Associations between time intervals and outcomes such as survival are often non-linear (U- or J-shaped) [10]. We assume similar findings apply to TTI. Particularly, the right slope of the U- or J-shape could demonstrate an increasing risk of poorer survival when patients encounter a longer TTI than the day at the nadir (the lowest point on the curve). The left slope could demonstrate the increased mortality risk when the subsequent treatment is initiated immediately; then, the risk would decrease in patients with a longer TTI (but less than the day at the nadir).

Regarding the impact of TTI on patient outcomes, several studies (including one RCT) in the settings of adjuvant chemotherapy or radiotherapy have been explicitly conducted, but most did not consider non-linear associations given the study design and analytic approach, treating the exposure TTI as a continuous or categorical variable in a traditional Cox-regression analysis [8, 9, 11–21]. However, a few studies had considered then demonstrated the association to be non-linear via a more advanced analytic method such as the restricted cubic spline model [17–21].

**Fig. 1:**
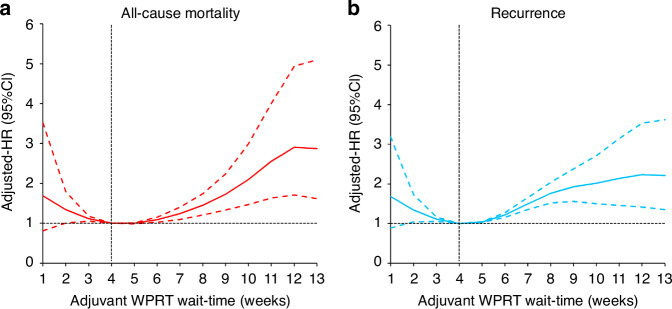
Non-linear association between time interval and patient outcome. Example of treatment-to-treatment interval (between surgery and adjuvant radiotherapy) associated with all-cause mortality (**a**) and disease-free survival (**b**) [21]. WPRT Whole pelvic radiotherapy, HR Hazard ratio, CI Confidence interval.

Below is an example of a study that demonstrated a U-shaped association. In brief, the study evaluated the impact of TTI between surgery (primary radical hysterectomy and pelvic lymphadenectomy) and adjuvant radiotherapy (whole pelvic irradiation) on all-cause mortality and disease-free survival (DFS) in 1541 women with clinical stage IB-IIB cervical cancer [21]. Based on a median of 5.6 years follow-up after surgery, the authors applied a Cox regression analysis with the restricted cubic spline model and found that patients with a TTI larger than six weeks had a higher risk of all-cause mortality (Fig. 1). Specifically, the adjusted hazard ratio was 1.45 (95%CI 1.21, 1.74) for week 8 and 2.91 (1.71, 4.95) for week 12. A similar pattern can be found in DFS. Given the pattern on the right slopes, the authors recommended adjuvant radiotherapy be administered within six weeks of surgery [21]. From the U-shaped association of all-cause mortality with the nadir at weeks 4–6, we can also see the pattern on the left slope: the risk of worse outcomes was increased right at the beginning when patients immediately received adjuvant radiotherapy after surgery; the risk started to decrease until reaching a minimum among patients with a larger TTI but less than 4–6 weeks (Fig. 1) [21].

Our assumptions on the U- or J-shaped associations were inspired by studies on traditional diagnostic and treatment intervals (e.g., the time between diagnosis and first treatment initiation). Many of these studies have debunked the typical assumption that prognosis worsens with a longer time in a linear association [10, 22–25]. Some have found non-linear associations, even U- or J-shape associations, relying on an analytic approach like the restricted cubic spline model to consider the non-linear pattern [10, 25–27].

Relying on the U- or J-shaped associations, an important indication for clinical and policy practice is that the pattern helps suggest the optimal timing between treatment modalities. Specifically, using the days at the nadir as the maximum timeframe [24], theoretically, patients would have a minimal mortality risk when receiving the subsequent treatment before the timeframe. At the same time, patients would potentially avoid an increased risk of mortality if they did not receive the subsequent treatment within a very short period of time after the former treatment.

In addition to the prognostic impact of TTI, studies can investigate the reasons or factors explaining the patterns in the U- or J- shape so that future interventions can address the barriers to treatment initiation within the optimal timing window. Particularly, studies can answer why patients have treatment delays with a longer TTI than the days at the nadir and why they have to immediately receive the subsequent treatment after the former treatment is discontinued.

To explain the U- or J- shaped associations, we assume medical, healthcare, and social rather than biological reasons and factors would play a significant role. To explain the pattern on the right side of the graph, studies explained that patients who delay receiving the subsequent treatment have a risk of experiencing a challenging course after the former treatment (e.g., complications, treatment toxicity, increased length of stay at the hospital, unplanned 30-day readmission) [7, 8, 10, 19, 28], and that these patients are more likely to have prognosis-related sociodemographic characteristics such female, Black, Asian and Hispanic races, older age, being uninsured, lower education and income levels, and being unmarried [7, 11, 14, 19, 28]. Of note, delay in subsequent treatment is common in real-world practice. For example, a study found over 90% of patients with stage II NSCLC received adjuvant chemotherapy outside of the 6-week timeframe usually used in RCTs [9]. In that study, reasons for delay included medical conditions like poor postoperative recovery (23%) and complications (16%), healthcare conditions like referral delay to medical oncology for treatment (16%), logistical delay due to treatment in remote areas (10%) and full re-staging before adjuvant chemotherapy as requested by the treating oncologists (8%), as well as social reasons like patient decision (18%) [9]. According to previous studies, reasons or factors for delays also include: 1) medical factors such as advanced stage [7, 11, 28], poorer performance status [11], comorbidities [7, 15, 20, 28], no completion of planned former treatment [15], type or regiment(s) of former treatment [2]; 2) healthcare factors such as long wait between the date of biomarker testing and the date of available results [29], delay in follow-up after the former treatment [8], academic or integrated network treatment facilities [7, 19] and treatments at more than one facility [7]; and 3) social factors such as long distance from treatment facility [7, 28] and states/regions for living [7, 28].

To explain the pattern on the left side of the graph, it is understandable that these patients with a higher mortality risk after immediately receiving the subsequent treatment may be yet to fully recover from the former treatment [12, 20]. This condition might partially explain a certain number of patients who failed to complete the subsequent treatment, as the main reasons include inability to tolerate the treatment (due to adverse events) and patient choice [8]. Of note, in the adjuvant setting, especially for advanced treatments such as antiangiogenic agents, an essential condition requiring patients to wait a certain time for the subsequent treatment is that surgical wound should be healed entirely; otherwise, patients who receive these treatments in the short term after surgery can have increased risks of surgical wound dehiscence, bleeding or infection, leading to treatment failure and potentially risking survival [20, 30, 31]. Among the patients who immediately receive subsequent treatment, some might be directly provided with the subsequent treatment like chemotherapy rather than taking time for a new pathological assessment so that a biomarker-driven treatment can possibly be eligible. This situation has been found in a certain proportion (22–24%) of patients with NSCLC [29].

Regarding biological reasons or factors accounting for the U- or J-shaped associations, we have not found strong evidence except for a few indications from previous studies [8, 9, 11–21]. For example, baseline results in one study show that patients who had estrogen receptor (ER) positive, progesterone receptor (PgR) positive, or HER2 negative early breast cancer were more likely to delay adjuvant chemotherapy [11]. Another study discussed immunosuppression induced by neoadjuvant treatments, which might play a role in mortality risk if patients have yet to recover and then shortly afterwards receive further treatments [20]. The study also discussed the downside of adjuvant radiotherapy as it could impair host immunologic effects, induce vascular damage, remodulate the tumour microenvironment, upregulate key molecules, and ultimately stimulate tumour invasion and metastasis [20, 32–34]. Another study reported that clinical practice has been shifting towards a longer TTI for oesophageal cancer surgery after neoadjuvant chemoradiotherapy, because of an increased probability of a complete histological response from neoadjuvant treatment [12, 35]. Confirmation and more discoveries are needed by future research.

### Other research focuses

In addition to TTI, we hold similar assumptions of the U- or J-shaped association of prognosis with the cycle-to-cycle interval of a treatment. Current evidence includes a lower survival probability in people with breast cancer who experienced inter-cycle delays of more than 7 days during the period of receiving neoadjuvant or adjuvant chemotherapy [36].

An area of focus could include the effect of treatment duration or discontinuation. Decisions on discontinuing treatments can be made for indications such as unacceptable or intolerable adverse events, disease progression or metastasis, recurrence, or patient choice (e.g., due to financial toxicity) [37, 38]. However, given that patients have significantly prolonged survival with advanced therapies, treatment discontinuation has become ambiguous, particularly when extending these treatments to the adjuvant setting for early-stage surgically treated cancers [2, 5, 6] and long-term use of immune checkpoint inhibitors [39–41]. Of note, even though the maximal duration of immunotherapies was two years (35 cycles) in clinical trials [39, 40], in the real world, patients can be on treatment continuously over the period [39–41], mainly given the impressive duration of treatment response (e.g., median of 23.2 months in nivolumab plus ipilimumab for advanced NSCLC [42]) so that medicine authorities like the U.S. Food and Drug Administration (FDA) and the European Medicines Agency (EMA) allow the continuation after two years. However, we have yet to see strong evidence of efficacious benefits from longer usage [39, 40], and there are concerns about health, financial, and societal toxicities [41]. These issues in treatment discontinuation reflect another under-researched area of focus – treatment deintensification or de-escalation [43, 44]. Studies should investigate the optimal duration of treatment that balances prognosis, quality of life, and financial costs to patients and the healthcare system.

Research on treatment timing may also include the order of advanced therapies before (neoadjuvant strategy), during (perioperative strategy), or after (adjuvant strategy) surgery or radiotherapy. Although several studies have started evaluating the prognostic impact of the treatment order, a clear, evidence-based consensus might yet be achieved [5]. For example, neoadjuvant versus adjuvant immunotherapy for NSCLC [5, 45], neoadjuvant versus adjuvant chemotherapy for early-stage breast cancer [46], and even the “sandwich approach” using treatment in both the neoadjuvant and adjuvant settings [47–49].

Of note, enriched treatment options and strategies require more considerate assessments in radiology and pathology and more frequent use of multidisciplinary meetings. Therefore, research is also needed to keep track of the length of traditional treatment intervals (including the period before the first treatment initiation), understand the current length of TTI between treatment lines, and evaluate the impact on patient experience and healthcare resourcing. We assume, given the enriched radiological and pathological assessments, the lengths of these time intervals might have yet to be improved, even longer than before. For example, a study reported a median of 21 days for biomarker testing results to be available after cancer diagnosis in patients who can receive first-line targeted therapies for advanced NSCLC [29]; the time period has been beyond the timeframe (14 days) of treatment interval between cancer diagnosis and treatment as recommended in international guidelines [50, 51]. We are concerned that many patients could still experience treatment delays [23]; at the same time, healthcare utilisation and expenditure may never decrease. These issues have been even of concern due to the COVID-19 pandemic period [52]. In addition to suggesting optimal timing and providing reasons or factors to explain the association patterns between timing and patient outcomes, research on the above focuses also brings implications of improving healthcare delivery through medical, healthcare and policy interventions.

## Opportunities for clinical trials and real-world studies

The above research questions and assumptions provide new opportunities for fostering evidence of the optimal timing of cancer treatments using the existing clinical trial and real-world data. Specific to the timing between treatments, we rarely see studies in advanced treatments like neoadjuvant/adjuvant targeted therapies or immunotherapies [8, 9, 11–21, 35].

As previously mentioned, current RCTs normally lack evaluation on the optimal timing of treatments; this might result from the restricted study designs influenced by the research questions from medicine authorities (e.g., FDA, EMA). Relying on the completed clinical trials, however, a post hoc analysis can be conducted if participants have varying lengths of treatment intervals or treatment duration. As these data had been obtained for drug approval, reusing them for post hoc analysis should be feasible and could provide direct insights into the optimal timing of treatments. Such an analysis might allow another opportunity for re-assessing treatment efficacy, especially for superiority trials that yield non-positive results based on the pre-specified design. At the same time, RCTs can bring better records of medical reasons for treatment delays or discontinuation (e.g., details of adverse effects and comorbidities) [12, 13, 16, 31, 38], which can explain the associations between treatment timing and patient outcomes.

Limitations of the study may include poor generalisability and potentially insufficient statistical power, given the nature of RCTs. Typically, due to the inclusion and exclusion criteria for recruiting patients, the RCT sample may not represent the whole patient population in the real world [53]. At the same time, the actual number of enroled patients in RCTs is subject to the pre-specified estimate for the minimum sample size for efficacy comparison [54], as well as difficulties in enrolment as reported with no more than half (49%) of enrolment success rate [55]. These conditions may render the post hoc analysis underpowered to estimate the optimal timing, especially in understanding the non-linear associations between TTI and patient outcomes and in analyses by various patient subgroups.

Using real-world data (RWD) from electronic health records or population-based registries can address the above limitations and may provide more relevant results. Real-world studies often provide a large and diverse study sample that typically represents the target real-world population, enabling investigation of the optimal timing with large statistical power and even allowing investigation of the potential reasons and factors accounting for treatment delays or discontinuation. Real-world studies could also provide insight into the impact on healthcare utilisation. In the real world, particularly, time intervals and treatment duration should be more variable as they are more impacted by multifaceted reasons or factors at the medical, healthcare, and social levels [56]; using RWD may be potentially more relevant to investigate optimal timing, typically, demonstrating and explaining the assumed U- or J-shaped associations between treatment timing and patient outcomes. However, study limitations include the lack of accurate recording of clinical information, difficulties in constructing study cohort and key variables if lacking multiple data sources and data linkage capacity, and challenges in addressing potential biases, given the nature of administrative or health electronic data that are not designed for research [57]. These limitations might have less impact if using hospital-level data with more detailed health records instead of population-level data.

Given the feasibility of using the current clinical trial data and RWD, we call for emerging evidence of the optimal timing of cancer treatments. We envision that various studies can be conducted, but the results might be specific, since the studies for this purpose can be context-specific based on study characteristics like particular treatment options, therapeutic settings, cancer types and biomarkers, healthcare settings, or systems. Given the large variations in study characteristics, we suggest careful study methodology and detailed reporting, allowing more cautious interpretation and comparative assessment in the future [1]. Particularly, we emphasise appropriate analytic approaches given the potential of non-linear associations between treatment intervals and patient outcomes, as assumed and carefully described above. Methodological frameworks and recommendations like the Aarhus statement are valuable to guide the research design and conduct [1, 24, 56, 58, 59], especially for traditional treatment intervals on which these recommendations mainly focus. In addition to using clinical trial data and RWD independently, further studies may utilise complementary features of both data sources to develop accurate and robust timing evaluations for a target patient population routinely seen in real-world clinical practice [60]. We anticipate that studies on the optimal timing will bring new insights into using cancer treatments better, even further improving treatment efficacy.
